# *Schistosoma mansoni* soluble egg antigen (SEA) and recombinant Omega-1 modulate induced CD4^+^ T-lymphocyte responses and HIV-1 infection *in vitro*

**DOI:** 10.1371/journal.ppat.1007924

**Published:** 2019-09-05

**Authors:** Emily EIM Mouser, Georgios Pollakis, Hermelijn H. Smits, Jordan Thomas, Maria Yazdanbakhsh, Esther C. de Jong, William A. Paxton

**Affiliations:** 1 Laboratory of Experimental Virology, Department of Medical Microbiology, Amsterdam UMC, Location Academic Medical Center, University of Amsterdam, Amsterdam, the Netherlands; 2 Department of Clinical Infection, Microbiology and Immunology (CIMI), Institute of Infection and Global Health, University of Liverpool, Liverpool, United Kingdom; 3 Department of Parasitology, Leiden University Medical Centre, Leiden, the Netherlands; 4 Department of Cell Biology and Histology, Amsterdam UMC, Location Academic Medical Center, Amsterdam, the Netherlands; 5 Department of Experimental Immunology, Amsterdam UMC, Location Academic Medical Center, Amsterdam, the Netherlands; Vaccine Research Center, UNITED STATES

## Abstract

Parasitic helminths evade, skew and dampen human immune responses through numerous mechanisms. Such effects will likely have consequences for HIV-1 transmission and disease progression. Here we analyzed the effects that soluble egg antigen (SEA) from *Schistosoma mansoni* had on modulating HIV-1 infection and cytokine/chemokine production *in vitro*. We determined that SEA, specifically through kappa-5, can potently bind to DC-SIGN and thereby blocks DC-SIGN mediated HIV-1 *trans*-infection (p<0.05) whilst not interfering with *cis*-infection. DCs exposed to SEA whilst maturing under Th2 promoting conditions, will upon co-culture with naïve T-cells induce a T-cell population that was less susceptible to HIV-1 R5 infection (p<0.05) compared to DCs unexposed to SEA, whereas HIV-1 X4 virus infection was unaffected. This was not observed for DCs exposed to SEA while maturing under Th1 or Th1/Th2 (T_mix_) promoting conditions. All T-cell populations induced by SEA exposed DCs demonstrate a reduced capacity to produce IFN-γ and MIP-1β. The infection profile of T-cells infected with HIV-1 R5 was not associated with down-modulation of CCR5 cell surface expression. We further show that DCs maturing under T_mix_ conditions exposed to plant recombinant omega-1 protein (rω-1), which demonstrates similar functions to natural ω-1, induced T-cell populations that were less sensitive for HIV-1 R5 infection (p<0.05), but not for X4 virus infection. This inhibition associated again with a reduction in IFN-γ and MIP-1β expression, but additionally correlated with reduced CCR5 expression. We have shown that SEA parasite antigens and more specifically rω-1 can modulate HIV-1 infectivity with the potential to influence disease course in co-infected individuals.

## Author summary

Parasitic helminths have developed a number of strategies to evade, skew and dampen human immune responses. Such effects will likely have consequences for HIV-1 transmission and disease progression. Here we analyzed the effect that soluble egg antigen (SEA) from *Schistosoma mansoni* had on HIV-1 infection *in vitro*. We determined that SEA, through kappa-5, can potently block DC-SIGN mediated HIV-1 trans-infection of CD4^*+*^ T-lymphocytes, but not block cis-infection. Dendritic cells (DC) exposed to SEA during maturation under Th2 skewing conditions, induce T-cell populations that are less susceptible to HIV-1 R5 infection compared to cells induced by unexposed DCs. HIV-1 X4 infection was unaffected. This restricted infection profile was not associated with down-modulation of CCR5 surface expression or observed differences in cytokine/chemokine production. Using recombinant omega-1, an abundant component of SEA, HIV-1 R5 infection was similarly inhibited with no effect on HIV-1 X4 infection levels. Hence SEA possesses antigens, namely omega-1, that can modulate HIV-1 infection and potentially influence disease course in co-infected individuals.

## Introduction

Humans encounter numerous pathogens throughout their life-time, encompassing bacteria, fungi, parasites and viruses with many infections occurring concomitantly. Since CD4^+^ T-lymphocytes are the main cell-type infected with human immunodeficiency virus type 1 (HIV-1), the immune responses mounted against the array of co-infecting pathogens will likely influence HIV-1 transmission and disease progression. Helminthic parasites such as *Schistosoma mansoni* (*S*. *mansoni*) are pertinent in this context, due to their ability to evade, dampen and skew the human immune system including the modulation of CD4^+^ T-lymphocyte responses. Moreover, many areas endemic for *S*. *mansoni* infection have high HIV-1 prevalence rates indicating that co-infection is likely.

Cells are infected with HIV-1 through the initial binding of its trimeric gp120 envelope protein to CD4, after which it interacts with numerous chemokine receptors, typically CCR5 or CXCR4, and undergoes entry [1]. CCR5 using viruses (R5) are those predominantly transmitted and later in disease in approximately 50% of individuals the virus switches to utilizing CXCR4 (X4) as a co-receptor [2]. Following transmission the virus rapidly disseminates to lymph nodes and especially to the gut associated lymphoid tissue (GALT). The GALT is a major reservoir for CD4^+^CCR5^+^ memory T-cells and approximately 80% of these cells are lost in the first weeks following HIV-1 infection [3,4]. Direct infection of cells via the CD4 molecule and co-receptors is termed *cis*-infection. An array of C-type lectins (CLR) expressed on myeloid cell lineages have been shown to successfully capture HIV-1 and pass the virus to activated CD4^+^ T-cells, referred to as *trans*-infection [5]. One such lectin known to strongly support *trans*-infection is dendritic cell specific ICAM3-grabbing non-integrin (DC-SIGN) which is expressed to high levels on dendritic cells (DCs). This molecule has been implicated in supporting HIV-1 transmission as well as virus dissemination [5,6]. DC-SIGN is known to bind many glycosylated structures including a large array of pathogen antigens as well as numerous host proteins found in bodily secretions [7–10]. Indeed, these molecules have the capacity to interfere with HIV-1 *trans*-infection.

CD4^+^ effector memory Thelper (Th) cells consist of three major subsets; Th1 cells induced by viral infections, Th2 cells induced by parasitic infections and Th17 cells induced by bacterial and fungal infections [11]. Remarkably, expression of HIV-1 co-receptors is not directly linked to the HIV-1 susceptibility of these cells. For instance, Th1 cells express high levels of CCR5 but also produce MIP-1α (CCL3), MIP-1β (CCL4) and RANTES (regulated upon activation normal T-cell expressed and secreted) (CCL5), the natural ligands for CCR5, thereby limiting R5 infection in these cultures [12,13]. Contrary to this, Th2 cells express lower levels of CCR5 but due to the limited production of MIP-1α, MIP-1β and RANTES these cultures have been shown to be infected with HIV-1 more easily [12,13]. This generalized view that Th2 cells are more susceptible to HIV-1 infection than Th1 cells is no longer supported. In a review by Mariana et al. it was stated that HIV-1 susceptibility of CD4^+^ T-cells varied dependent on the *in vitro* stimulation of these cells [14]. More recent studies have correlated pathogen specific CD4^+^ T-cell phenotypes to HIV-1 susceptibility. Cytomegalovirus (CMV) and *Mycobacterium tuberculosis* (*Mtb*) infections both result in the induction of Th1 cells [15,16]. However, the *Mtb* specific T-cells are lost early during HIV-1 infection while the CMV specific T-cells are lost later in disease [17]. This discrepancy was explained by differences in cytokine expression profiles, where *Mtb* specific cells possess a high IL-2 and low MIP-1β expression pattern, the reverse phenotype was observed in CMV specific CD4^+^ T-cells [17]. Human papilloma virus specific CD4^+^ T-lymphocytes have also been shown to be lost early after HIV-1 infection [18,19].

Helminths, including *S*. *mansoni*, are known to skew immune responses towards a Th2 phenotype, which according to the above hypothesis would be detrimental to those individuals co-infected with HIV-1 [20,21]. This has led to the assumption that treating *S*. *mansoni* in co-infected individuals would be beneficial for their HIV-1 disease. Clear epidemiological evidence to-date is lacking, as treatment studies have been reporting contradictory findings [22]. A treatment program in Ethiopia found that deworming *S*. *mansoni* infected HIV-1 patients led to a decrease in viral loads [23], whilst another study in Uganda reported the opposite [24]. Similar inconsistencies have been found for other markers associated with HIV-1 disease progression as reviewed in [21], with only one exception. Women infected with *S*. *haematobium* and who have egg induced lesions in their genital tract were found to be at higher risk of HIV-1 infection [25,26].

In *S*. *mansoni* infections the eggs play a crucial role in disease as they induce lesions and skew CD4^+^ T-lymphocyte responses. An adult *S*. *mansoni* pair typically lay up to 300 eggs a day which migrate to the gut lumen in order to be expelled [27]. One of the best studied antigen mixtures of *S*. *manoni* is soluble egg antigen (SEA) which is an extract derived from homogenized eggs and consists of hundreds of proteins of which many are glycosylated [28]. SEA has accordingly been shown to bind many glycan receptors including DC-SIGN, mannose receptor (MR) and macrophage galactose type-lectin (MGL) [28,29]. Through binding to these receptors SEA alters the DCs response to TLR4 ligand, LPS (lipopolysaccharide) and TLR3 ligand PolyI:C [30]. Although, SEA itself cannot fully mature immature DC (iDCs), while antigen processing is similar to LPS matured DCs [31]. Furthermore, SEA exposed DCs are known to induce Th-cell responses that are skewed towards a Th2 phenotype, even when a Th1 cell response is required [32]. Recently it has been demonstrated that omega-1 (ω-1), one of SEA’s main components, is able to drive Th2 cell responses [33–35]. Omega-1 is a member of the T2 RNase family which enters the cell through binding MR and subsequently degrades cellular mRNA and rRNA products. Both the RNase activity and the glycan group are essential for Th2 skewing [33].

Infection with either *S*. *mansoni* or HIV-1 has major implications for the host due to the longevity of infection and extent of damage these pathogens cause to the immune system. The complex pathogen interactions encountered in co-infected individuals makes it difficult to determine the effect of *S*. *mansoni* on HIV-1 infection. Consequently, our study focused on the effects of SEA on HIV-1 infection, where we specifically address whether SEA can interfere with *cis*- or *trans*-infection of CD4^+^ T-lymphocytes as well as whether the effects exerted by SEA on DC maturation can modulate the T-cell population’s susceptibility to HIV-1 infection.

## Results

### SEA inhibits HIV-1 *trans*-infection but not cis-infection of CD4^+^ T-lymphocytes

**Fig 1:**
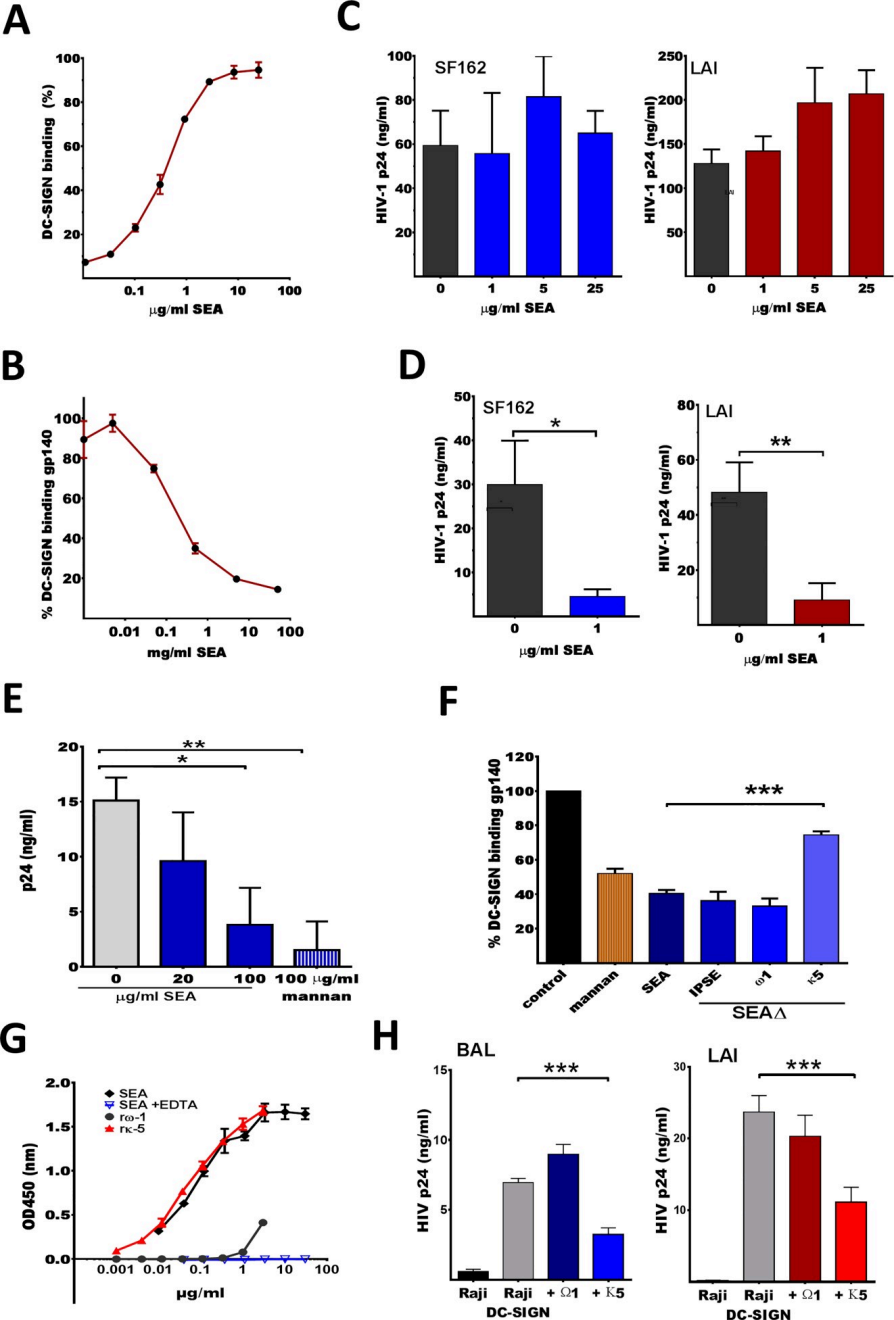
SEA binds DC-SIGN and can block *trans*-infection of CD4 T-lymphocytes but not *cis*-infection. **(A)** DC-SIGN binding ELISA where DC-SIGN-Fc was added to 5 fold serial dilutions of SEA coated onto an ELISA plate, the highest OD_450_ was set to 100% binding with ±SEM shown. **(B)** A gp140 competition ELISA where DC-SIGN-Fc was incubated with serial dilutions of SEA for 30min at RT prior to addition to a trimeric gp140 coated ELISA plate (captured via an HIV-1 Env antibody). The highest OD_450_ was set to 100%, implying that 0.01μg/ml SEA will not block DC-SIGN-Fc binding to gp140. **(C)** Effect of SEA on *cis*-infection was determined by adding SEA 2h prior to SF162 (R5) or LAI (X4) to CD4^+^ enriched T-lymphocytes. Viral outgrowth was monitored through measuring p24 levels in culture supernatant at either day 5 or 7 of infection. **(D)** SEA’s effect on *trans*-infection was determined by pre-incubating Raji DC-SIGN cells with 1μg/ml SEA for 2h and subsequently adding SF162 (TCID_50_ 5000) or LAI (TCID_50_ 500). Viral outgrowth in Raji DC-SIGN—CD4^+^ T-lymphocyte co-cultures was monitored through measuring p24 levels in culture supernatant. **(E)** Similarly, iDC were incubated with SEA or mannan (positive control) for 30min prior to addition of SF162 (TCID_50_ 200). Viral outgrowth in iDC—CD4^+^ T-lymphocyte co-cultures was monitored through measuring p24 levels in culture supernatant at either day 5 or 7 of infection. **(F)** SEA components rκ5 (1.7μg/ml), IPSE or rω-1 (both 2μg/ml) were examined in the gp140 competition ELISA. The OD_450_ of DC-SIGN-Fc binding to gp140 is set to 100% and binding of SEA pre-incubated DC-SIGN-Fc is plotted as a percentage of this 100%. Pre-incubating DC-SIGN-Fc with any of the SEA fractions or mannan resulted in a significant decrease (p<0.05) in gp140 binding. **(G)** SEA as well as rω-1 or rκ5 were coated at limiting dilution on an ELISA plate and analyzed for DC-SIGN-Fc binding. **(H)** Recombinant protein effects on *trans*-infection were determined by pre-incubating Raji DC-SIGN cells with rω-1 (10 µg/ml) or rκ5 (10 µg/ml) for 2h and subsequently adding BAL (TCID_50_/ml 1000) or LAI (TCID_50_/ml) virus followed by enriched CD4^+^ lymphocytes and measuring p24 levels in culture supernatant on day 5. (**B-H**) Experiments were performed in triplicate and data shown as mean of these triplicates with ±SD. *p< 0.05,**p<0.01, ***p<0.001.

It has been reported that SEA binds several C-type lectin receptors and competes with monomeric HIV-1 gp120 for binding [28,36], but has not been tested for inhibiting HIV-1 capture and transfer. We confirmed that our derived SEA binds DC-SIGN using a DC-SIGN binding ELISA, where increasing concentrations of SEA results in a dose-dependent increase of DC-SIGN-Fc binding (Fig 1A). To determine whether this interaction interferes with HIV-1 binding to DC-SIGN we performed a gp140 competition ELISA. Here DC-SIGN-Fc is incubated with SEA before addition to an ELISA plate coated with trimeric gp140. We found that concentrations as low as 0.2μg/ml SEA resulted in a 50% reduction of DC-SIGN-Fc binding to gp140 (Fig 1B). Since the trimeric gp140 protein closely resembles the functional HIV-1 envelope protein, this data suggests that SEA can prevent HIV-1 from interacting with DC-SIGN.

We next tested whether SEA could interfere with HIV-1 *cis*-infection of CD4^+^ lymphocytes (direct infection). Therefore, CD4^+^ enriched (CD8^+^ depleted) T-cell blasts were incubated with SEA (1, 5 or 25μg/ml) before HIV-1 SF162 (CCR5 using virus) or LAI (CXCR4 using virus) was added. Neither SF162 (R5) nor LAI (X4) viral outgrowth, measured as the concentration of HIV-1 capsid protein (CA-p24) in the culture supernatant, was affected by the presence of SEA (Fig 1C). This indicates that SEA does not interfere with HIV-1 binding to CD4, CCR5 or CXCR4. Additionally, the presence of SEA had no visible effect on cell counts and/or cell viability of the CD4^+^ enriched T-cells.

Since incubating DC-SIGN-Fc with 1μg/ml SEA provided a 70% reduction in its capacity to bind gp140 (Fig 1B), we pre-incubated Raji DC-SIGN cells with the same concentration to identify whether SEA can block HIV-1 *trans*-infection of CD4^+^ T-cells. Viral outgrowth of both SF162 (R5) and LAI (X4) was approximately 80% reduced in co-cultures where the Raji DC-SIGN cells were pre-incubated with SEA (p<0.01 and p<0.05, respectively) (Fig 1D). Similarly, we tested the effect of SEA on immature monocyte derived DCs (iDCs) and observed a significant reduction (p<0.01) in SF162 (R5) outgrowth but only when iDCs were pre-incubated with 100μg/ml SEA (Fig 1E). This higher SEA concentration was likely required because iDCs express higher levels of DC-SIGN than Raji DC-SIGN cells, have multiple other CLRs capable of binding HIV-1 and have a rapid receptor turnover. Mannan, known to bind DC-SIGN and block HIV-1 *trans-*infection, was tested as a positive control and provided similar inhibitions (p<0.001).

SEA consists of many molecules of which IPSE/α-1, kappa-5 (κ5) and omega-1 (ω-1) are the major components. To determine whether any of these could be responsible for blocking HIV-1 *trans-*infection, SEA depleted of each product was evaluated using the gp140 competition ELISA. Depletion of κ5 from SEA partially restored the binding capacity of DC-SIGN to gp140 (p<0.001) whereas depletion of IPSE/α-1 or ω-1 did not abrogate the effect (Fig 1F). We next tested whether plant derived recombinant omega-1 (rω-1) or recombinant κ5 (rκ5), which carry similar glycosylation profiling as natural derived products [37], could bind to DC-SIGN in the standard binding ELISA assay. SEA showed a dose dependent binding to coated DC-SIGN as did rκ5, whilst rω-1 demonstrated no binding, supporting the previous finding where depletion of natural derived κ5 from SEA removed DC-SIGN binding activity (Fig 1G). Furthermore, when testing rω-1 and rκ5 in a Raji-DC-SIGN mediated capture transfer experiment, only rκ5 inhibited HIV-1 infection of CD4^+^ T-lymphocytes (p<0.001) (Fig 1H).

In conclusion, SEA potently binds DC-SIGN, via κ5, and prevents DC-SIGN mediated capture and transfer of HIV-1 to CD4^+^ T-lymphocytes, whilst having no effect on direct infection of CD4^+^ lymphocytes.

### SEA exposed DCs induce T-cell cultures with an altered susceptibility to R5 HIV-1 infection

**Fig 2:**
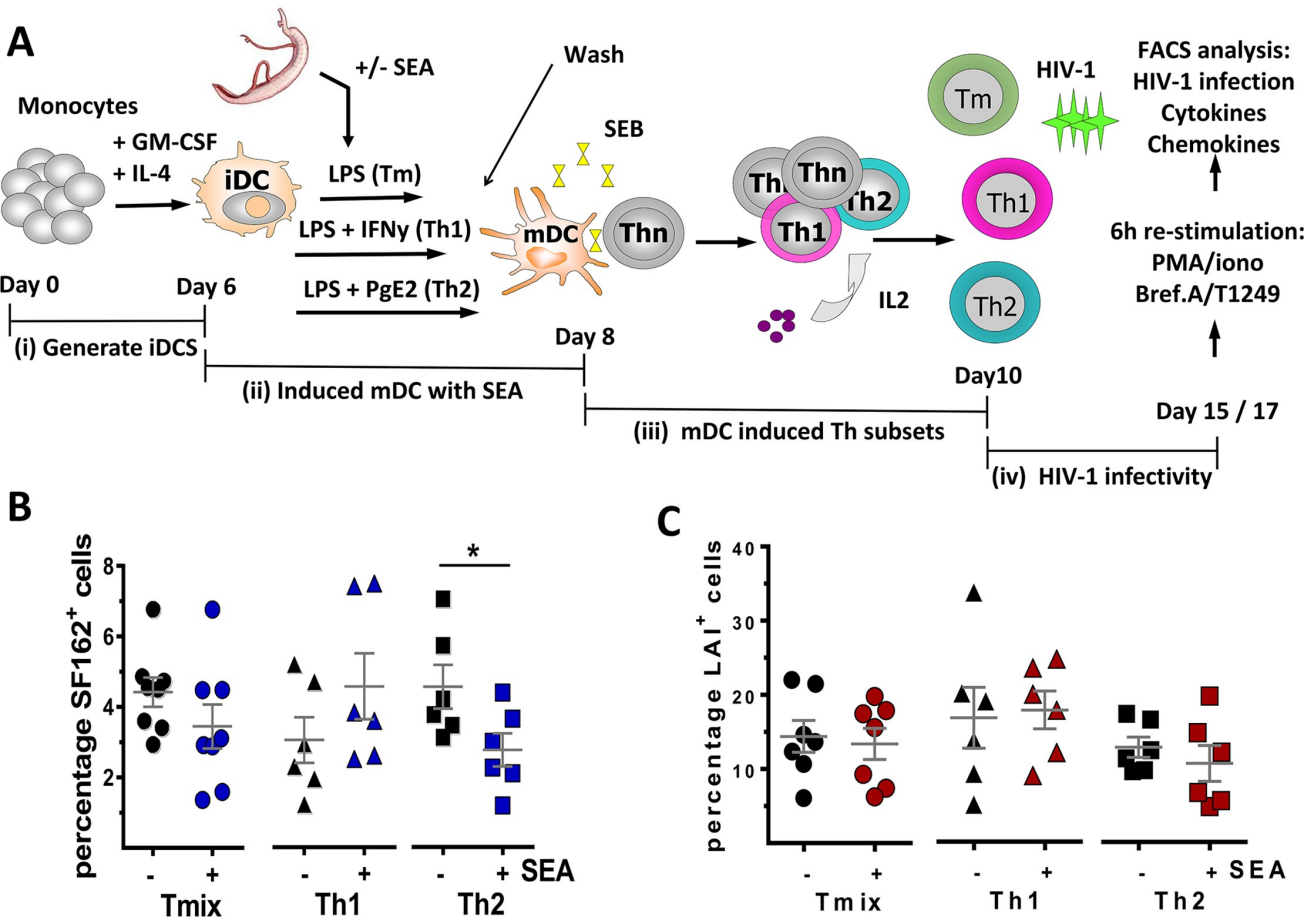
HIV-1 SF162 (R5) infection is altered in T-cell cultures induced by SEA exposed DCs. **(A)** Schematic overview of our *in vitro* model system. Isolated iDCs are matured under T_mix_, Th1 or Th2 promoting conditions either in absence or presence of parasite antigen. Subsequently, the matured DCs were co-cultured with naïve CD4^+^ T-cells in combination with Staphylococcal Enterotoxin B (SEB). The induced effector memory T-cell populations (T_mix_, Th1 or Th2 cell cultures) were then infected with HIV-1 after which, among others, the percentage of HIV-1 infected cells was measured. **(B)** Percentage of SF162^+^ cells in T_mix_, Th1 and Th2 cell cultures induced by DCs unexposed (black) or exposed to SEA (blue). **(C)** Percentage of LAI^+^ cells in T_mix_, Th1 and Th2 cell cultures induced by DCs unexposed (black) or exposed to SEA (red). (**B and C**) Results of 6 to 8 independent experiments using at least 5 different donors combined and depict are the mean value and SEM. *p<0.05.

Besides the direct effect of blocking HIV-1 *trans*-infection, SEA may also affect HIV-1 infection indirectly. SEA exposed DCs have been shown to promote the development of T-cells that are skewed towards a Th2 phenotype [32]. Since it has been shown that differentially skewed Th cell populations have variant HIV-1 infection profiles [12,13], it is likely that SEA can modulate HIV-1 infection and/or replication. Here we aimed to i) determine whether the presence of SEA during DC maturation alters the polarizing capacity resulting in a modified Th cell profile and ii) identify whether such a modulated Th cell phenotype has an altered susceptibility for HIV-1. We therefore established an *in vitro* model system where we could assess the Th cell populations, induced by DCs matured in the absence or presence of parasite products, for cytokine/chemokine production and HIV-1 infection (Schematic shown Fig 2A). Here iDCs (donor A) were matured in Th1/Th2 mixed (T_mix_)-, Th1- or Th2-promoting conditions (LPS, LPS+IFN-γ or LPS+PgE_2,_ respectively) either in the absence or presence of SEA. Subsequently, the matured DCs were washed, to remove the SEA, and co-cultured with naïve CD4^+^CD45RA^+^ T-cells (donor B) and Staphylococcus Enterotoxin B (SEB) for 8 days resulting in memory Th cell populations with specific phenotypes. These cells were then infected with HIV-1 SF162 (R5) or LAI (X4) and monitored for infection over time (Fig 2A and S1 Fig). In our model, Th cell populations induced by DC matured under T_mix_ and Th2 promoting conditions harbour on average 4.4% ±0.4 and 4.6% ±0.6 SF162^+^ cells, respectively, whilst Th cells induced by DCs matured under Th1 promoting conditions have a lower level of infection, 3.1% ±0.7 (p = 0.09, compared to T_mix_ cell culture) (Fig 2B, black symbols). When SEA was present during DC maturation the percentage of SF162^+^ T-cells was found to be similar in T_mix_, Th1 and Th2 cell cultures, 3.5% ±0.6, 4.6% ±0.9 and 2.8% ±0.5, respectively (Fig 2B, blue symbols). A 2-way ANOVA revealed that there is a significant interaction (p = 0.041) between SEA and the Th cell subset infected with SF162, meaning that SEA affects SF162 infection depending on the Th cell subset. More in depth analysis of the different groups demonstrated that a Th2 cell population induced by SEA exposed DCs have a significantly lower percentage of SF162^+^ T-cells than a Th2 cell population induced by SEA unexposed DCs (p = 0.044). In contrast, SEA exposure of DCs inducing T_mix_ or Th1 populations did not affect the percentage of SF162^+^ T-cells.

No such effect was observed in LAI (X4) infected Th cell populations. The percentage of LAI^+^ cells in Th cell cultures induced by DCs, matured under T_mix_, Th1 and Th2 promoting conditions, were all found to be similar, 14.4% ±2.2, 16.7% ±4.1 and 12.8% ±1.4, respectively, in the absence of SEA or 13.5% ±2.1, 17.8% ±2.5 and 10.6% ±2.4, respectively, in the presence of SEA (Fig 2C). Analysing the effect of SEA on infection of different Th cell populations using a 2-way ANOVA demonstrated no significant differences. Our data demonstrate that the addition of SEA to DCs maturing under Th2 promoting conditions result in the induction of a Th cell population with a reduced susceptibility to HIV-1 (R5) infection.

### Reduced susceptibility of CD4^+^ T-lymphocytes to HIV-1 (R5) via SEA exposed DCs does not correlate to altered cytokine/chemokine expression levels

**Fig 3:**
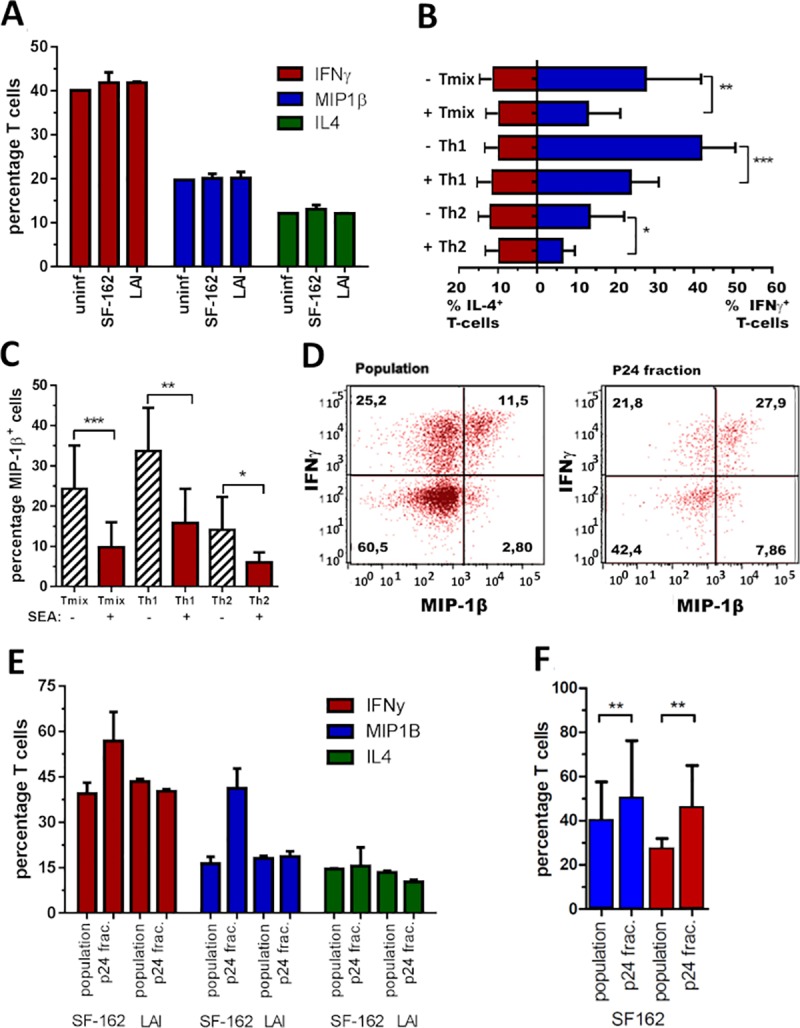
DCs exposed to SEA, induce T-cell cultures with less IFN-γ and MIP-1β producing T-cells. **(A)** Percentage of Tmix-cells producing IFN-γ, MIP-1β and IL-4 in an uninfected (bar 1), SF162 (bar 2) or LAI (bar 3) infected T-cell population., Representative for T_mix_, Th1 and Th2 cell cultures induced by DCs matured either in absence or presence of SEA. **(B)** Plotted is the average percentage of IL-4 and IFN-γ producing T-cells in T_mix_, Th1 and Th2 cell cultures induced by DCs matured either in absence or presence of SEA. **(C)** Average percentage of MIP-1β producing T-cells in the three T-cell populations induced by DCs matured in absence or presence of SEA. **(D)** Dot-plot of IFN-γ (y-axis) and MIP-1β (x-axis) producing Th1 cells in the SF162 population (left) and the p24^+^ fraction of that population (right) shown in the next panel. **(E)** Percentage of IFN-γ, MIP-1β and IL-4 producing T-cells in a SF162 or LAI infected Th1 cell culture (bar 1 and 3) versus the percentage found in the p24^+^ fraction (bar 2 and 4). **(F)** Data of 7 independent experiments combined depicting the percentage of IFN-γ and MIP-1β producing T-cells in a SF162 infected Th1 cell population versus the percentage found in the p24^+^ fraction of that population. Both Figs (**E** and **F**) are representative for all three T-cell populations induced by DCs matured either in absence or presence of SEA. **(A, D, E)** Representative figures from at least 5 experiments (conditions with virus were performed in duplicate) while **(B, C, F)** are the results of 6 to 8 independent experiments using at least 5 donor combinations combined, depict are the mean value and SD. *p<0.05 **p<0.01 ***p<0.001.

In order to explain the reduced SF162 (R5) infection in Th2 cell cultures induced by SEA exposed DCs we characterized the cytokine/chemokine (IFN-γ, IL-4, IL-2, TNF-α, MIP-1β) expression profiles of the different Th cell populations. First we demonstrated that neither SF162 (R5) nor LAI (X4) infection alters the potential of the T-cells to produce cytokines in T_mix_ cells (Fig 3A). Next, we determined the effect of exposing DCs to SEA on Th cell responses and found a significant reduction of IFN-γ producing T-cells in all three conditions irrespective of HIV-1 infection (Fig 3B). Although IL-4 production of the T-cells remained similar, the ratio, IL-4/ IFN-γ did increase when T-cells were induced by DCs matured in the presence of SEA (Fig 3B and S1 Table). Besides IFN-γ, the percentage of MIP-1β^+^ cells was also significantly reduced in all three T-cell populations where SEA had been present during DC maturation (Fig 3C).

Interestingly, when comparing the cytokine profile of the SF162 infected (p24^+^) T-cells to the profile of the total population we found a higher percentage of IFN-γ and MIP-1β producing T-cells in the infected fraction (for all three T-cell conditions) (Fig 3D and 3E). Analysing the FACS plots revealed it is mainly the percentage of MIP-1β^+^ and IFN-γ^+^MIP-1β^+^ cells that is enhanced (Fig 3D). Combining data from several donors shows a significant increase in MIP-1β^+^ and IFN-γ^+^ cells in the p24^+^ fraction (p<0.05) (Fig 3F). This increase was not observed for IL-4 nor for any cytokine monitored in the LAI (X4) infected fractions (Fig 3E). However, since this increase in MIP-1β^+^ and IFN-γ^+^ cells in the SF162 (R5) infected fraction is found in all three T-cell populations induced by SEA exposed DCs, it is unlikely that this phenomena contributes to the altered SF162 (R5) susceptibility of Th2 cell cultures induced by SEA exposed DCs. Hence, the cytokine/chemokine production of the T-cells does not explain the significant reduction in SF162 (R5) infection found in Th2 cell cultures induced by DCs exposed to SEA. Nevertheless, the high percentage of MIP-1β producing T-cells in Th1 cell cultures induced by unexposed DCs may explain the trend of reduced SF162 infection levels in this population (p = 0.09) (Fig 2B).

### Reduced susceptibility of CD4^+^ T-lymphocytes to HIV-1 (R5) does not correlate with altered CCR5 expression

**Fig 4:**
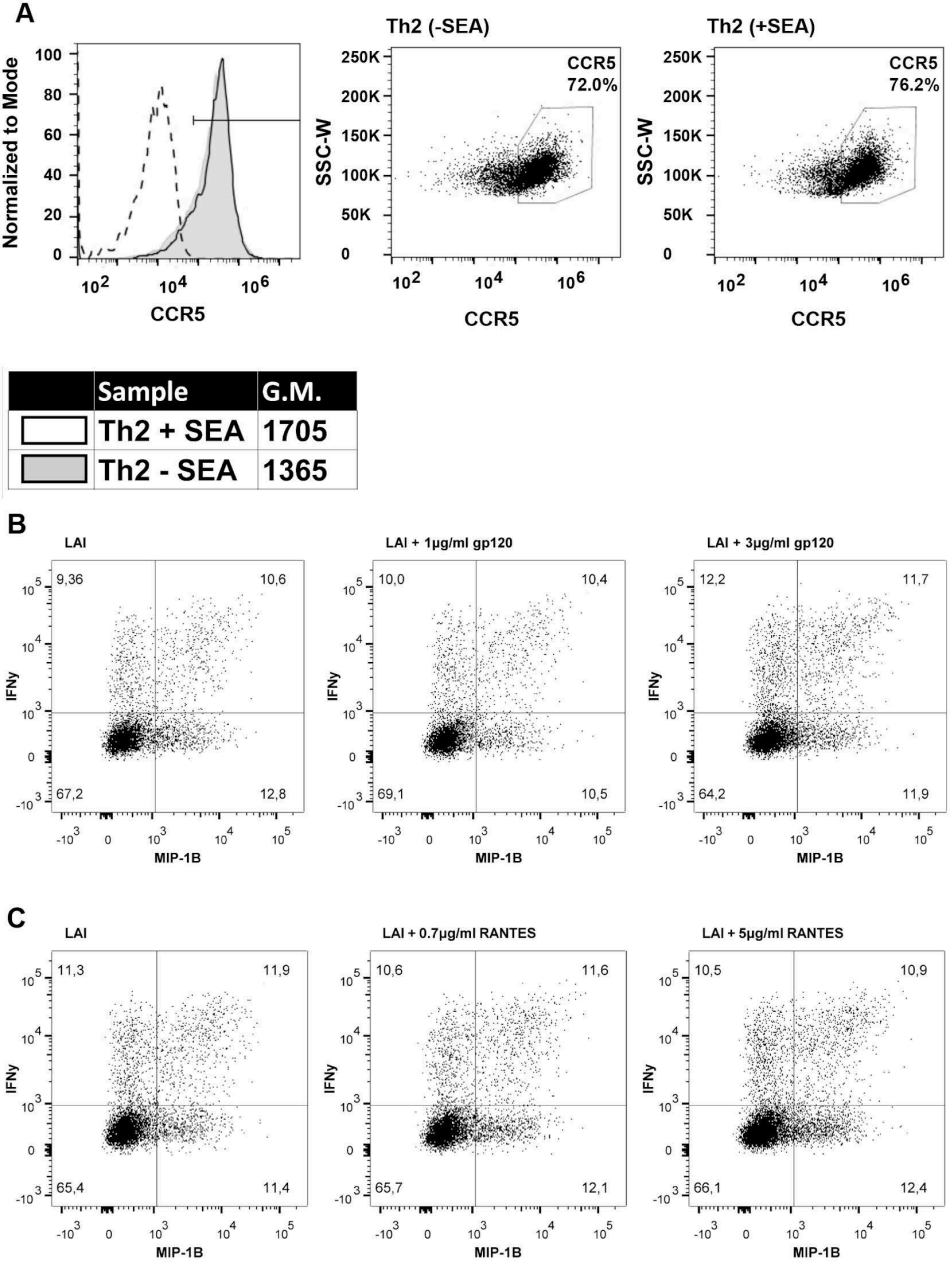
CCR5 expression does not correlate with altered SF162 infection. **(A)** Th2 cell cultures induced by DCs matured in absence or presence of SEA (dotted line unstained control), analyzed for the percentage of cells with high levels of CCR5 on their surface as well as the quantity of CCR5 on the surface of each cell (geometric mean). **(B)** Dot-plots showing the percentage of IFN-γ (y-axis) and MIP-1β (x-axis) producing T-cells in the LAI^+^ fraction of LAI infected cultures (left), LAI infected cultures with 1μg/ml (middle) or 3μg/ml (right) monomeric SF162 gp120 added 24h after LAI infection. **(C)** Dotplots showing percentage of IFN-γ (y-axis) and MIP-1β (x-axis) producing T-cells in the LAI^+^ fraction of LAI infected cultures (left), LAI infected cultures with 0.7μg/ml (middle) or 5μg/ml (right) RANTES added 24h after LAI infection. **(A-C)** Are representative figures of experiments performed at least 3 times.

The reduced HIV-1 infection was only observed for SF162 (R5) and not for LAI (X4), suggesting that CCR5 expression levels may be altered in the Th cell populations induced by SEA exposed DCs. We determined the percentage of cells expressing CCR5 on the surface as well as the quantity of CCR5 per cell. When comparing the Th2 cell cultures induced by DCs, either exposed or unexposed to SEA, we found no difference in the percentage of cells with a high expression of CCR5, 72% versus 76.6% respectively, as determined by the gate based on the marker or the amount of CCR5 expressed on each cell, geometric mean of 1365 and 1705, respectively (Fig 4A). The interesting finding that the SF162- but not LAI-infected fraction had a significantly higher percentage of IFN-γ^+^ and MIP-1β^+^ T-cells than the total population (Fig 3E) led us to speculate that SF162 (R5) infection resulted in upregulation of these cytokines through CCR5 engagement rather than the specific cell phenotype being targeted by SF162 (R5) virus. To examine this possibility we added either monomeric SF162 gp120 or RANTES (a CCR5 ligand) to LAI (X4) infected cultures and determined the percentage of IFN-γ and MIP-1β producing T-cells. Addition of monomeric SF162 gp120 (1 and 3μg/ml) or RANTES (0.7 and 5μg/ml) did not increase the percentage of IFN-γ^+^ or MIP-1β^+^ T-cells compared to 9.3% and 12.8% found in the LAI infected fraction in the absence of these compounds (Fig 4B and 4C). Our data indicates that CCR5 signalling is not the cause of heightened IFN-γ^+^ and MIP-1β^+^ T-cells in the SF162^+^ fraction. To conclude, we found no difference in CCR5 expression between Th2 cell cultures induced by DCs, either exposed or unexposed to SEA. Furthermore, it is unlikely that signalling through CCR5 causes the increased percentage of IFN-γ and MIP-1β producing T-cells in the SF162 infected fraction compared to the total population.

### Recombinant ω-1 (rω-1) exposed DCs induce T_mix_ cell cultures with a reduced R5 HIV-1 susceptibility

**Fig 5:**
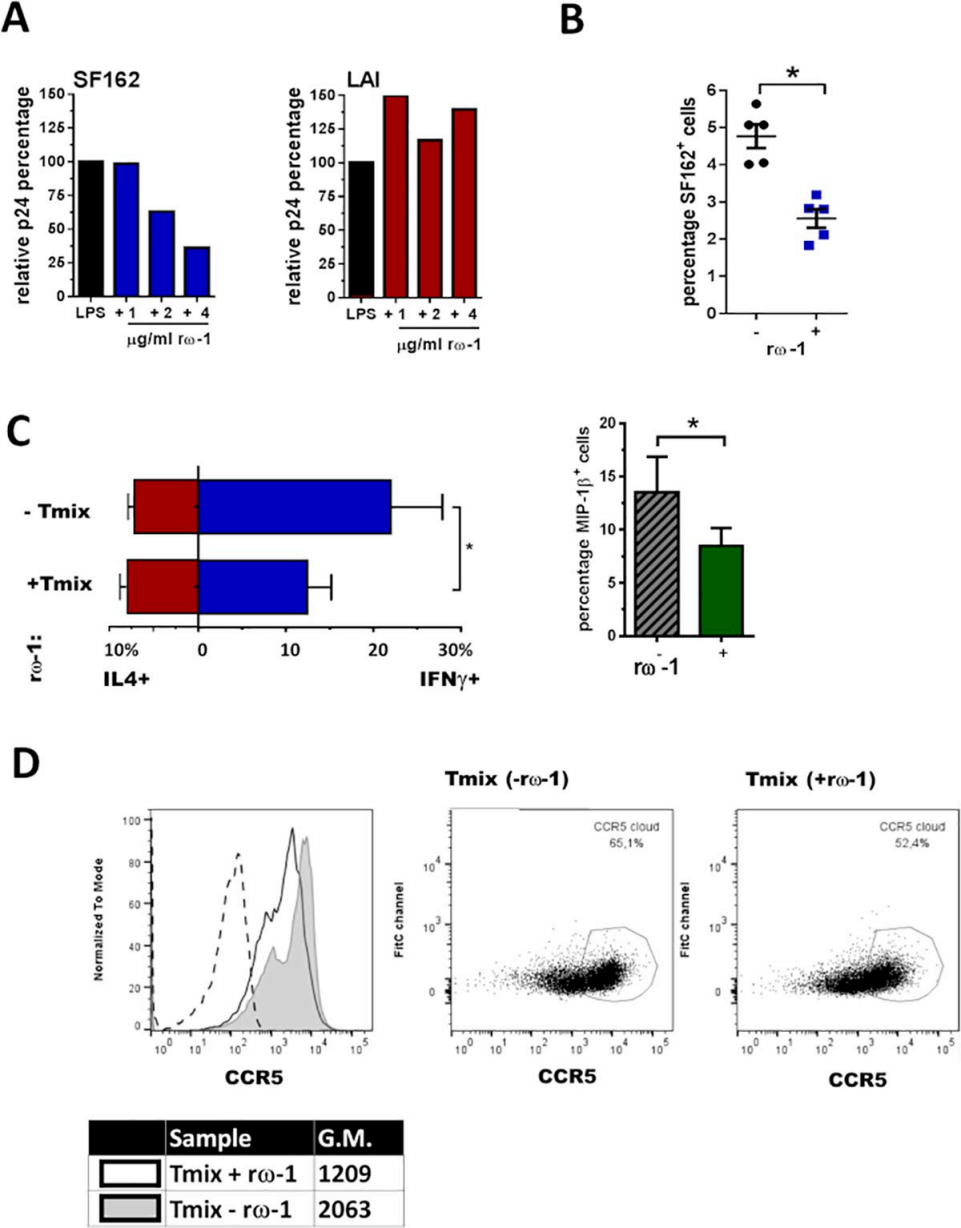
DCs exposed to rω-1 induce a T_mix_ cell population less susceptible to SF162 (R5) but not LAI (X4) infection. (**A**) Percentage of SF162 or LAI infected cells in T_mix_ cell populations induced by rω-1 unexposed DCs was set to 100% and the level of infection found in T_mix_ cell cultures induced by rω-1 exposed DCs was expressed as a percentage of this 100%. (**B**) The percentage of SF162^+^ cells found in T_mix_ cell populations induced by unexposed DCs versus the percentage found T_mix_ cell populations induced by rω-1 exposed DCs (left panel) with +/- SE shown, with the same shown for LAI (right panel) with +/- SE depict. (**C**) Average percentage (± SEM) of IL-4, IFN-γ and MIP-1β producing T-cells in T_mix_ cell cultures induced by unexposed or rω-1 exposed DCs and depict with SD. (**D**) Percentage of cells with high CCR5 expression on their surface as well as the quantity of CCR5 per cell (geometric mean) for T_mix_ cell cultures induced by unexposed or rω-1 exposed DCs (dotted line unstained control), representative figure of 3 independent experiments. (**B, C**) Data from four independent experiments (± SD) using four different donor combinations where DCs were matured in the presence of 2–4μg/ml rω-1. *p<0.05.

We have demonstrated that Th2 cell cultures induced with SEA exposed DCs harbour a significantly lower percentage of SF162 infected cells compared to Th2 cell cultures induced by unexposed DCs. Since the T_mix_ cell population is more relevant to the *in vivo* scenario and where Th1 and Th2 cross-talk likely resides to influence responses we were interested to follow this further. Indeed, T_mix_ cell culture SF162 infection levels were lower (although not statistically significant) when DCs were exposed to SEA (Fig 2B). Since omega-1 has been described as the main Th2 skewing component of SEA we aimed to study the effect of its recombinant form (rω-1) on the different cell subsets [33,34,37]. In an initial experiment we identified that the addition of rω-1 (3µg/ml) in the T_mix_ and Th2, but not the Th1, setting reduced infection with SF162 virus (S2A Fig). When focussing further on the T_mix_ population we identified that the presence of 2µg/ml or 4µg/ml, but not 1µg/ml), rω-1 during DC maturation resulted in T_mix_ cell cultures with a reduced susceptibility for SF162 (R5) infection, 40% and 60% less infected, respectively (Fig 5A). Moreover, the presence of rω-1 during DC maturation did not affect the induced T-cell population’s susceptibility to LAI (X4) virus. Plotting data from independent experiments (n = 5) confirms that there is significantly less infection with SF162 (R5) virus in T_mix_ cell cultures induced by DCs exposed to rω-1 during maturation, whilst not being observed with LAI (X4) infections (Fig 5B).

Analysing the cytokine/chemokine profile of T_mix_ cell populations induced by rω-1 exposed DCs resulted in a similar pattern as observed for T-cell populations induced by SEA exposed DCs. There was a significant reduction in the percentage of IFN-γ and MIP-1β producing T-cells whilst the percentage of IL-4 producing cells remained unaltered (Fig 5C). Based on the cytokine/chemokine profile of these cells it does not appear that rω-1 leads to enhanced Th2 skewing. What we did find was a modest, 10% reduction in the percentage T-cells with a high expression of CCR5 (indicated by the gate based on the marker) in T_mix_ cell cultures induced by rω-1 exposed DCs. Similarly, we observed a reduced level of CCR5 surface expression per cell, geometric mean of 1,209 versus 2,063, respectively which potentially plays a role in the reduced infection with R5 virus (Fig 5D). Collectively, these results indicate that ω-1 is the component responsible for the reduced SF162 (R5) infection observed in Th2 cell populations induced by SEA exposed DCs and that the molecule can similarly reduce infection of T_mix_ cells at higher concentrations.

## Discussion

Helminthic parasites are known to possess immunomodulatory properties and specifically skew immune responses towards a Th2 phenotype [32]. Here we studied the effect’s SEA has on modulating HIV-1 infection *in vitro*. Although SEA did not affect *cis*-infection of CD4^+^ enriched T-cell blasts, it efficiently blocked DC-SIGN mediated HIV-1 *trans*-infection through the binding of Kappa-5 to DC-SIGN. We demonstrated that exposing DCs maturing under Th2 promoting conditions to SEA induce Th cells that were less susceptible to R5 but not X4 infection and that rω-1 possessed the same activity with DCs maturing under T_mix_ promoting conditions. Collectively, our data demonstrates that SEA has the capacity to influence numerous mechanisms associated with HIV-1 transmission and pathogenesis, suggesting that the *S*. *mansoni* infection has the potential to modulate HIV-1 infection as well as disease course.

Receptive anal intercourse carries the highest high risk for HIV-1 transmission, likely due to the nature of the mucosal barrier and the immune cells residing at this site [2,38]. DCs will be amongst the first cells encountering HIV-1 with the potential of transferring virus to CD4^+^ T-lymphocytes either locally or in adjacent lymph nodes [5]. The presence of SEA in the gut wall may therefore influence *trans*-infection and interfere with the transmission process. Similarly, Th cell responses induced in localised lymph nodes by DCs exposed to SEA, more so ω-1, could have a phenotype that is less susceptible to infection with R5 HIV-1, again limiting the likelihood of transmission [39]. Moreover, regardless of the route of HIV-1 infection, a massive loss of CD4^+^ memory T-lymphocytes in the GALT is observed within the first few weeks of infection [4]. This reservoir is not replenished after peak viremia and although the initial cell loss is not correlated with HIV-1 disease progression, microbial translocation caused by loss of the GALTs function is [4,40]. The potential reduction in HIV-1 infection of GALT tissue exposed to SEA/ω-1 may therefore reduce not just cellular infection profiles but also the effects of bacterial translocation.

Several cytokines and CC-chemokines have been associated with alterations in HIV-1 infection and disease progression. MIP-1α, MIP-1β and RANTES are the natural ligands for CCR5, hence *in vitro* T-cell cultures containing high levels of these chemokines have lower levels of HIV-1 R5 infection [41,42] as has been observed *ex vivo* [17]. In contrast, we observed a significantly lower percentage of Th cells capable of producing IFN-γ and MIP-1β when they were induced by DCs matured under T_mix_, Th1 or Th2 promoting conditions in the presence of SEA or rω-1 (Figs 3C and 5C). The HIV-1 R5 infected fraction however, had a higher percentage of Th cells producing IFN-γ and MIP-1β then the total population. This suggests either that these cells were targeted or that IFN-γ and MIP-1β were induced upon HIV-1 R5 infection. Addition of monomeric HIV-1 R5 gp120 or recombinant RANTES to HIV-1 X4 infected cell cultures did not induce a similar effect, suggesting the latter is not the case. Targeting of these cells seems equally unlikely since MIP-1β competes with HIV-1 R5 for CCR5 binding [12,42–44]. HIV-1 R5 may induce IFN-γ and MIP-1β via another mechanism, for example some forms of the viral protein Nef induce MIP-1β production in macrophages [45,46] while the viral protein Tat can induce MIP-1β secretion by neural progenitor cells [47]. Although it may seem counter intuitive that HIV-1 infection stimulates expression of MIP-1β, the function of this chemokine is to recruit CD4^+^ T-lymphocytes to the site of infection, thereby enhancing the number of target cells for HIV-1 replication [48]. It has also been shown that IL-2^+^ expressing cells are rapidly lost as a consequence of HIV-1 infection due to high levels of infection [17,49]. Since IL-2 is added to our cell culture system to ensure T-cell survival, we are unable to analyze the effects of IL-2 on HIV-1 infection with our system.

Besides cytokines and chemokines, we explored whether differences in surface expression levels of CCR5 could explain reduced infection with HIV-1 R5 but not X4. In Th2 cell cultures induced by SEA exposed DCs we found similar percentages of cells expressing CCR5 and similar levels of CCR5 on the surface of each cell (geometric mean) compared to Th2 cell cultures induced by unexposed DCs. As observed with SEA, rω-1 also reduced the DCs capacity to produce cytokines and co-stimulatory molecules in response to stimulus [33–35]. The mechanism via which a Th2 response is induced is still unknown, with one hypothesis being that the lack of stimuli provided by the DCs to the naïve T cells pushes them into default mode, namely the Th2 phenotype [35,50,51]. Yet, rω-1 exposed DCs induced T_mix_ cell cultures with even lower levels of HIV-1 R5 infection showed a reduced percentage of cells expressing CCR5 as well as a lower level of CCR5 expression per cell. Consequently, although CCR5 is not the main mechanism responsible for reduction in HIV-1 R5 infection it will likely contribute to the overall reduction of infection observed in T_mix_ cell cultures induced by DCs exposed to rω-1.

There are several hypotheses to explain the reduced infection of CD4^+^ T-lymphocytes, one of which is the capacity of SEA and rω-1 exposed DCs to induce regulatory T (Treg) cells [52,53]. Treg cells are susceptible to HIV-1 although several studies demonstrate that infection is restricted. It has been demonstrated that Treg cells were less susceptible to HIV-1 (R5) infection than effector memory T-lymphocytes while X4 viruses gave higher or similar levels of infection [54]. Further comparisons revealed these cells expressed similar levels of CD4, CCR5 and CXCR4 on their surface and produced similar levels of CC-chemokine production. Although no explanation was provided, the cellular activation status as well as FoxP3 interference patterns were thought to play a role. Some studies support the notion that FoxP3 inhibits HIV-1 infection by interfering with HIV-1’s LTR transcription activation [55,56] while others demonstrate enhanced HIV-1 production in FoxP3 positive cells [57,58]. The role of FoxP3 seems to be dependent on the viral strain as well as the culture protocol used for the cells. Another hypothesis is that virus production may be limited but not susceptibility [59], or that viral restriction factors, such as APOBEC3G may limit infectivity [60]. Further phenotypic characterisation of T-cells induced by SEA exposed DCs and more specifically rω-1 will undoubtedly identify additional cellular differentiation markers that associate with HIV-1 infection and/or replication with potential implications for induction of viral latency. A more in-depth analysis of SEA and rω-1 alterations to expression patterns of specific transcription factors associated with Th cell differentiation will further highlight the mechanisms responsible and associate such differences with the altered cytokine/chemokine expression patterns.

As well as SEA and rω-1 driving the induction of Th2 phenotypes [32–35], SEA has been shown to down-modulate DC TLR4 as well as LPS induced signalling [30]. More in-depth analysis with human DCs has demonstrated that SEA exposed DCs have enhanced expression of the negative regulators SOCS1 and SHP1 which result in impaired DC maturation and induction of CD4^+^ lymphocyte proliferation [61]. Similarly, in a murine model system it has been shown that SEA treated DC possess impaired LPS mediated maturation as identified through reduced expression of co-stimulatory molecules [62]. Our results support these findings and indicate that SEA/rω-1, through interacting with DCs, can induce CD4^+^ lymphocytes responses with reduced proliferative/activation phenotypes with potential consequences for HIV-1 infection and/or replication.

Reduced infection of CD4^+^ T-lymphocytes with R5 but not X4 HIV-1 may restrict initial viral replication or slow the rate at which HIV-1 switches its co-receptor phenotype. Large scale monitoring of study cohorts would need to be performed to identify whether such effects indeed do exist and which would be limited by factors such as parasite load and corresponding egg count. This complexity is confirmed by the discrepancies found in epidemiological studies of co-infected individuals [21,22]. The findings that HIV-1 R5 viruses replicated better in Th2 than Th1 cells resulted in the interpretation that treating *S*. *mansoni* infection may benefit co-infected patients [20,21]. Whether this can correlate to the *in vivo* setting needs to be determined and as always with such findings, antigenic concentration and the localisation of the source will be instrumental in determining to what capacity co-infection is beneficial. The effects of helminthic infections have been shown to dampen immune inflammatory responses with considerable consequences for diseases associated with such effects [63]. This would suggest that SEA antigens, including κ5 and ω-1, possess the physiological capacity to influence HIV-1 infection and disease. These skewing effects must ultimately be considered in the context of parasite antigens having the capacity to activate immune responses which provides a complex balance where different antigens have variant effects within co-infected individuals. We provide evidence here that Th cells induced by SEA/rω-1 exposed DCs are less susceptible to R5 HIV-1 infection, suggesting that helminthic infections may be beneficial when considering HIV-1. Deciphering the mechanisms may provide a means towards modulating immune responses beneficial for limiting viral transmission or reducing viral loads. Furthermore, these results should be considered in the context of HIV-1 vaccine trials being conducted in regions of the world where *S*. *mansoni* infections are endemic.

## Materials and methods

### Parasitic products

SEA was prepared and isolated as described previously [32]. Kappa-5 (κ5) was isolated by soybean agglutinin (SBA; Sigma, Zwijndrecht, the Netherlands) affinity chromatography as described previously [64]. The remaining SEA fraction was labeled as κ5 depleted SEA. Omega-1 and IPSE/alpha-1 were purified from SEA via cation exchange chromatography as previously described [35,49,65]. Omega-1 was then separated from IPSE/alpha-1 by affinity chromatography using specific anti-IPSE/alpha-1 monoclonal antibodies coupled to an NHS-HiTrap Sepharose column according to the manufacturer's instructions (GE Healthcare). Purified components were concentrated and dialyzed. Omega-1–depleted SEA or IPSE-depleted SEA were prepared by adding back purified IPSE/alpha-1 or omega-1, respectively, to the remaining SEA fraction left from the cation exchange chromatography. The purity of the preparations was controlled by SDS-PAGE and silver staining. Protein concentrations were tested using the Bradford or BCA procedure [66]. Plant produced rω-1 and rκ5 were purified from apoplast fluid using HS POROS 50 strong cation exchange (CEX) resin (Applied Biosystems) as previously described [34]. Apoplast fluids were transferred over G25 Sephadex columns to exchange for CEX binding buffer (20 mM phosphate buffered saline (pH 6.0) containing 100 mM NaCl). The plant derived rω-1 and rκ-5 molecule is similar to helminthic derived proteins with similar post-translational modifications, specifically glycosylation profiling, as observed in parasites.

### Viruses

SF162 (R5), BAL (R5) and LAI (X4) are replication competent HIV-1 subtype B viruses. SF162and BAL are molecular cloned isolates obtained from HIV-1 infected patients and which utilise CCR5 as co-receptor. LAI represents a molecular cloned virus isolated from an HIV-1 infected patient and utilises the CXCR4 co-receptor for infection. Viruses were passaged on CD4^+^ enriched T lymphocytes and tissue culture infectious dose (TCID_50_) values were determined by limiting dilutions on CD4^+^ enriched T-lymphocytes according to the Reed and Muench method, as previously described [67].

### ELISA assays

The DC-SIGN binding ELISA was performed as described [10]. Briefly, the components of interest (SEA, rω-1 or rκ5) were coated at 5 fold dilutions (50μg/ml– 0.01μg/ml on an ELISA plate after which 333ng/ml DC-SIGN-Fc (R&D systems) was added. Subsequently, DC-SIGN-Fc was detected by a secondary goat-anti-human-Fc HRP labelled antibody (Jackson Immunology), diluted 1:1000 and using standard ELISA procedures. In the gp140 competition ELISA, 10μg/ml anti-HIV-1 gp120 antibody D7324 (Aalto BioReagents Ltd) was coated on an ELISA plate after which trimeric HIV-1 gp140 (JR-FL SOSIP.R6-IZ-D7324) was added to the plate as previously described [68,69]. Meanwhile, 333ng/ml DC-SIGN-Fc (R&D systems) was incubated with SEA at limiting dilutions. Next, the mixture was added to the gp140 coated plate and using a secondary HRP labelled goat-anti-human-Fc antibody (Jackson Immunology) diluted at 1:1000, DC-SIGN-Fc binding to the gp140 plate was determined. A more detailed description can be found [68]. The capsid p24 ELISA was performed as standard [8]. In short, an ELISA plate was coated O/N with 10#x00B5;g/ml sheep anti-p24-specific antibody (Aalto Bio Reagents Ltd.). Culture supernatant was added, followed by 4ng/ml mouse anti-HIV-1-p24 alkaline phosphatase conjugate antibody (Aalto Bio Reagents Ltd.). Development solution Lumi-phos plus (Lumigen Inc.) was used according to the manufacturer's protocol. The standard curve consists of a serial dilution of *Escherichia coli*-expressed recombinant HIV-1-p24 (Aalto Bio Reagents Ltd.).

### Cell lines and primary cell isolation

Raji DC-SIGN cells (a kind gift from Prof TBH Geijtenbeek, University of Amsterdam), an immortalized B cell line stably transfected with DC-SIGN [5] was cultured in RPMI 1640 (Invitrogen) supplemented with 10% FCS, 100U/ml penicillin and 100U/ml streptomycin. Peripheral blood mononuclear cells (PBMCs) were isolated using ficoll-hypaque density centrifugation from buffy coats purchased from the Netherlands Blood bank (Sanquin). PBMCs of three CCR5 wild-type homozygous donors were pooled and cultured in RPMI 1640 containing 10% FCS, 100U/ml penicillin, 100U/ml streptomycin, 100U/ml recombinant IL-2 and cells were activated with 2µg/ml phytohemagglutinin (PHA) (Sigma). To enrich for CD4^+^ T-lymphocytes, the CD8^+^ T-lymphocyte population was depleted using CD8 dynabeads (Life Technologies) according to manufacturer’s protocol at day 5. These cells were used for experiments depict (Fig 1), as for the remaining experiments cells were isolated from fresh blood.

Monocytes were isolated from fresh blood using lymphoprep (Nycomed) and subsequent percoll (GE Healthcare) density gradient (34, 47.5 and 60% of standard isotone percoll) centrifugation. For 6 days monocytes were cultured in IMDM (Gibco) containing 5% FCS, 86μg/ml gentamycin (Duchefa), 500U/ml GM-CSF (Schering-Plough) and 10U/ml IL-4 (Miltenyi) differentiating them into immature DCs (iDCs). From the PBMC fraction the CD4^+^ T-cells were isolated using the CD4 T-cell isolation MACS kit (Miltenyi Biotec., 130-091-155) according to manufacturer’s protocol. Subsequently, the CD4^+^CD45RA^+^CD45RO^-^ naïve T-cells were isolated from the CD4^+^ T-lymphocytes using anti-CD45RO-PE (DAKO, R084301) and anti-PE beads (Miltenyi-Biotec, 130-048-801), described in detail [70].

### HIV-1 cis-infection assay

CD4^+^ enriched T-cells (2x10^5^ cells/well) were incubated with 25, 5 or 1μg/ml SEA or medium (control) for 2h in a 96-well flat-bottomed culture plate (Greiner Bio-One), subsequently medium, SF-162 (TCID_50_/ml of 200) or LAI (TCID_50_/ml of 200) was added to the well. At day’s 4, 7 and 12 supernatants were harvested and cells were fed with fresh media. HIV-1 capsid p24 was determined in the supernatants using a standard ELISA protocol.

### HIV-1 trans-infection assay

Raji DC-SIGN cells (5x10^4^cells/well) were pre-incubated with 1μg/ml SEA for 2h after which SF162 (R5) or LAI (X4) viruses were added at an end concentration of 200 TCID_50_/ml. After a further 2h incubation the cells were washed 3 times with PBS and co-cultured with enriched CD4^+^ T-lymphocytes (2x10^5^ cells/well). Viral outgrowth was measured by monitoring capsid p24 at day 4, 7 and 12 in harvested culture supernatant. HIV-1 capture/transfer by iDCs was conducted in a similar manner with minor modifications; 20 or 100μg/ml SEA was utilized and the incubation steps were shortened to 30min.

### T-lymphocyte outgrowth model system

A co-culture system was developed where monocytes were pre-incubated with immune skewing reagents in the presence or absence of parasite product (SEA or rω-1), before washing and adding to isolated memory T-cells and subsequently monitoring HIV-1 infection profiles or markers of CD4 cell phenotype. Routinely, iDCs from donor A were matured for 2 days with 100ng/ml LPS (Sigma-Aldrich) (which typically generates DCs that induce a Th_mix_-cell phenotypes), 100ng/ml LPS and 100U/ml IFN-γ (UCytech) (which typically generates DCs that induce a Th1-cell phenotype) or 100ng/ml LPS and 10μM PgE_2_ (Sigma-Aldrich) (which typically generates DCs that induce a Th2-cell phenotype) either in the absence or presence of 25μg/ml SEA or 1–4μg/ml rω-1. After thoroughly washing the matured DCs 3 times, 5x10^3^ of cells were co-cultured with 2x10^4^ CD4^+^CD45RA^+^CD45RO^-^ T-cells (naive) from donor B in 96-well flat bottom culture plates and 10pg/ml Staphylococcus enterotoxin B (SEB) (Sigma-Aldrich) in IMDM, 10% FCS, 86μg/ml gentamycin (Duchefa). The addition of SEB in combination with allogeneic stimulation of cells provides for maximal T-cell stimulation and proliferation in order to best achieve the numbers required for analysis. Cells were split at day 5 and day 7 with 20U/ml IL-2 being added to the medium. At day 8, 5x10^4^ cells/well were plated on 96-well flat bottomed plates and infected with SF162 (TCID_50_/ml 1000) or LAI (TCID_50_/ml 200). Since iDCs don’t survive this length of time in culture under these conditions the measured levels of infection represent direct *cis*-infection of Th-cells, indeed monitoring cell phenotypes indicated that only CD4^+^ lymphocytes were present at time of infection. Day 5 and 7 post-infection cells were re-stimulated for 6h with 10ng/ml PMA (Sigma-Aldrich), 1μg/ml ionomycin (Sigma-Aldrich), 10μg/ml brefeldin A (Sigma-Aldrich) supplemented with 0.1μg/ml T1294 (Pepscan Therapeutics BV) to prevent new infections during this period. Subsequently, cells were fixed in 3.7% formaldehyde and stored for no more than 1 week at 4⁰C in FACS buffer (PBS+ 2%FCS) for flow cytometry analysis measuring intracellular viral p24 antigen as well as an array of cell surface markers or intracellular cytokines/chemokines. Where monomeric gp120 (SF162) (Immune Technology Corp.) or RANTES (Sigma-Aldrich) were added the experiments were performed similarly. These compounds (concentrations in text) were added to the T-cell culture 24h after LAI infection and four days later the cells were re-stimulated. Experiments with SEA show the results of at least five different donor combinations and for experiments using rω-1 four different donor combinations are depicted in figures (see respective Fig legends).

### Flow cytometry

To measure intracellular activation markers, cells were permeabilized for 30min with PermWash at RT (BD Pharmingen) and subsequently stained with p24-PE (Beckman Coulter) and IFN-γ-FitC, IL2-PerCp-Cy5.5, IL-4-APC, TNF-α-PE-CF594, Mip-1β-AlexaFluor700 (all from BD Bioscience) for 30min at 4°C at a pre-determined dilution. Next, these cells were washed once with PermWash and taken up in FACS buffer (PBS+ 2%FCS) after which they were analyzed using a FACS Canto II machine. For determining CCR5 surface expression, T-cells obtained from our T-cell outgrowth model were fixed in 3.7% formaldehyde before HIV-1 infection and stored for no more than 1 week at 4°C in FACS buffer. These cells were stained in FACS buffer with CCR5 PE-Cy7 (Biolegend) for 30min at RT, washed with FACS buffer and measured. Shown in histograms are cytokine producing T-cells with samples taken 5 and 7 days after HIV-1 infection with the optimal time point being determined by the percentage of live cells (>50%) and level of HIV-1 infection, which varied per donor and per virus. Gating strategies and flow cytometric controls are represented (S3A Fig and S3B Fig, respectively).

### Ethics statement

All cells were isolated from anonymized buffy coats purchased through the Nederlands Blood Bank (Sanquin) where IRB approval was granted and donors signed a specifically consented in the additional use of samples for research purposes as outlined in the research contract between the AMC and Sanquin (number NVT0202.02).

### Statistical analysis

Data was analyzed using an unpaired t-test when comparing two groups and a 1-way ANOVA when comparing several groups (Fig 1). For the remaining figures the data is corrected for the systematic differences between donors using factor correction [71]. Subsequently, within each specific T-cell population the effect of induction with SEA/rω-1 exposed or unexposed DCs was compared using a paired T-test whereas data between two T-cell population’s data was compared using an unpaired T-test. Additionally for Fig 2, a 2-way ANOVA was performed to determine whether there was an interaction between the T-cell population infected and the usage of SEA.

## Supporting information

S1 Fig Th1/Th2 ratio in the different T-cell cultures. Depicted are dot-plots showing the percentages of IL-4 (y-axis) and IFN- (x-axis) producing T-cells in the T_mix_ (left), Th1 (middle) and Th2 (right) cell cultures of a typical experiment.
(TIF)

S2 Fig Effect of rω-1 exposed DCs on modulating HIV-1 infection of CD4 differing T-cell populations. (**A**) Tmix, Th1 and Th2 cells induced in the absence (dark blue) or presence (light blue) of rω-1 (3µg/ml) infected with HIV-1 SF162 (R5) virus and measured as p24% positivity (**B**) Tmix, Th1 and Th2 cells induced in the absence (dark red) or presence (light red) of rω-1 (3µg/ml) infected with HIV-1 LAI (R5) virus and measured as p24% positivity.
(TIF)

S3 Fig Gating strategy and control staining for p24^+^ cells and intracellular cytokine stainings. (**A**) Gating of p24^+^ cells was performed using a live cell gate using FSC and SSC (left panel), a single cell gate using FSC width (middle panel) and a p24^+^ cell gate (right panel). (**B**) Single staining’s for IFNγ (left), IL-4 (2^nd^ left), MIP-1α (3^rd^ left) and p24 (right) of T cells re-stimulated with PMA and ionomycin for 6hrs in the presence of Brefeldin A. Markers are set on positive cells and used for subsequent analysis of T-cell phenotype.
(TIF)

S1 Table Ratio of IL-4 / IFN-γ in various cell cultures. Here the ratio of IL-4 and IFN-γ for each cell culture induced DCs matured in the absence or presence of SEA is demonstrated.
(PPTX)
