# Supplementary figures and images for: Schistosoma mansoni soluble egg antigen (SEA) and recombinant Omega-1 modulate induced CD4+ T-lymphocyte responses and HIV-1 infection in vitro
Source: PLoS Pathog. 2019 Sep 5;15(9):e1007924. doi: 10.1371/journal.ppat.1007924 (PMC6728022; doi:10.1371/journal.ppat.1007924)

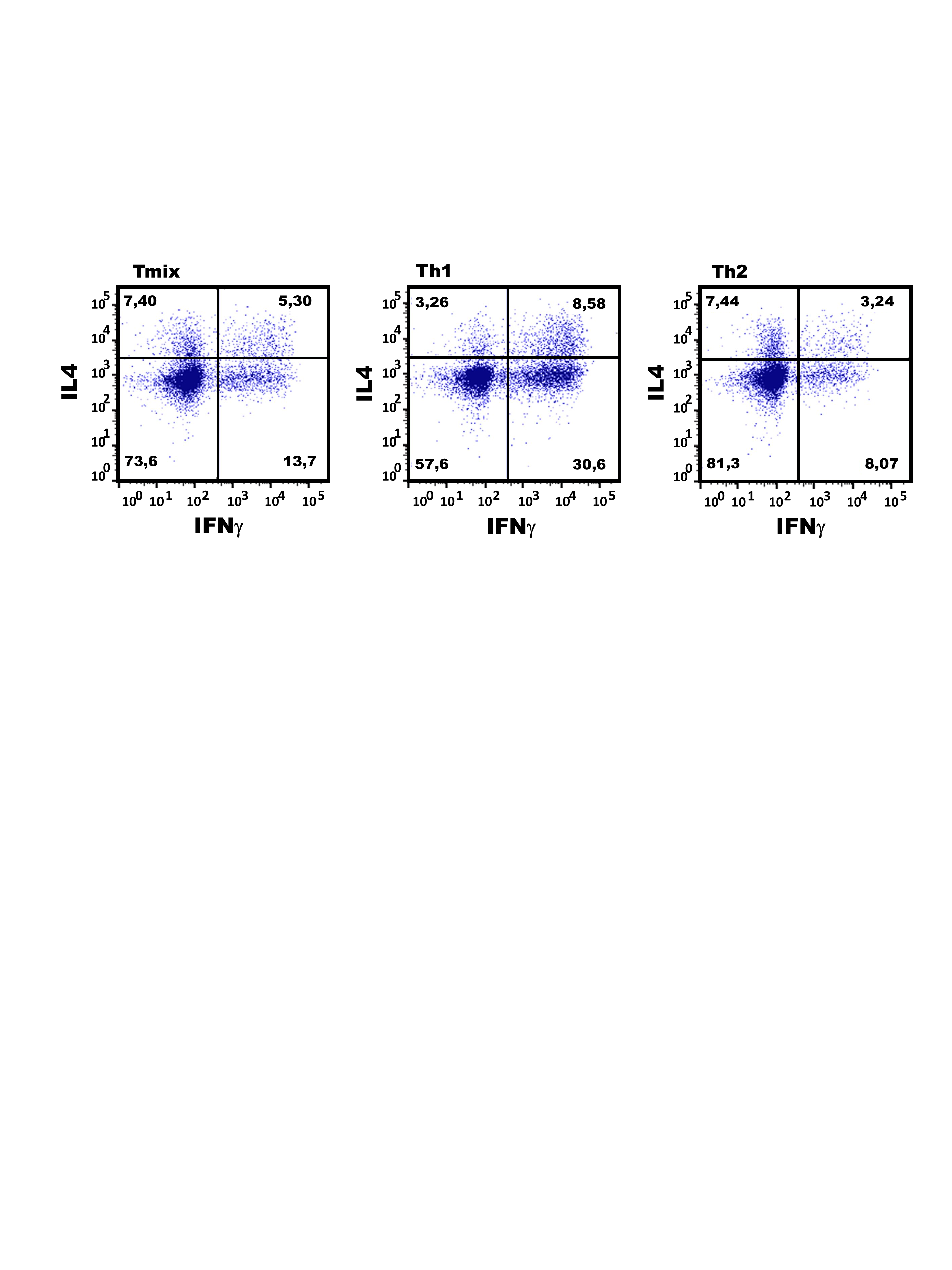

Supplement: S1 Fig — Depicted are dot-plots showing the percentages of IL-4 (y-axis) and IFN- (x-axis) producing T-cells in the Tmix (left), Th1 (middle) and Th2 (right) cell cultures of a typical experiment. (TIF) [file ppat.1007924.s001.tif]

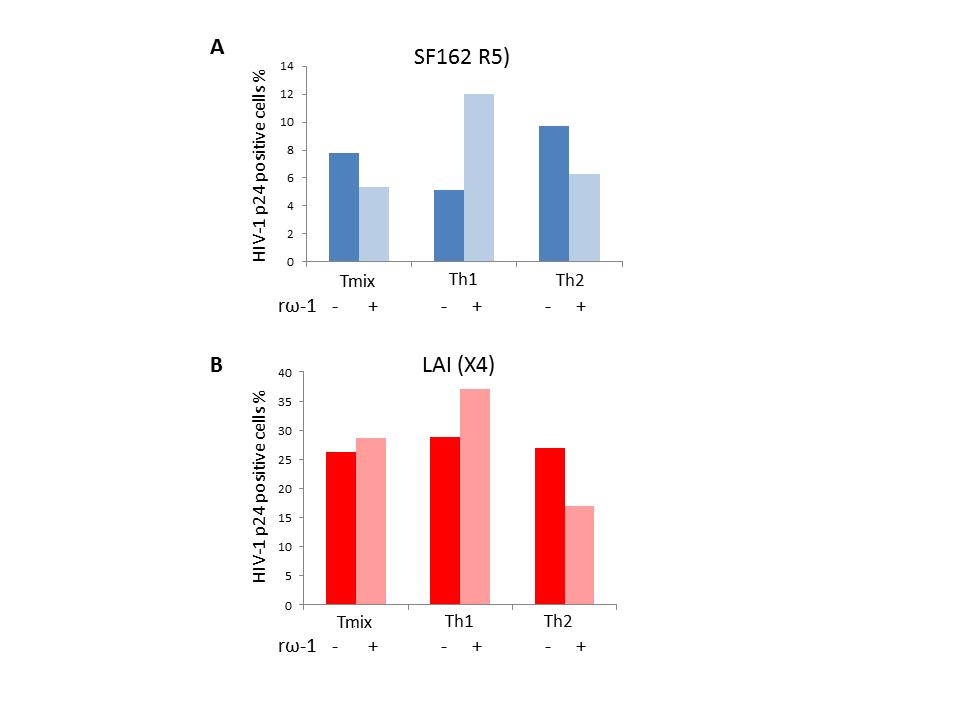

Supplement: S2 Fig — (A) Tmix, Th1 and Th2 cells induced in the absence (dark blue) or presence (light blue) of rω-1 (3µg/ml) infected with HIV-1 SF162 (R5) virus and measured as p24% positivity (B) Tmix, Th1 and Th2 cells induced in the absence (dark red) or presence (light red) of rω-1 (3µg/ml) infected with HIV-1 LAI (R5) virus and measured as p24% positivity. (TIF) [file ppat.1007924.s002.tif]

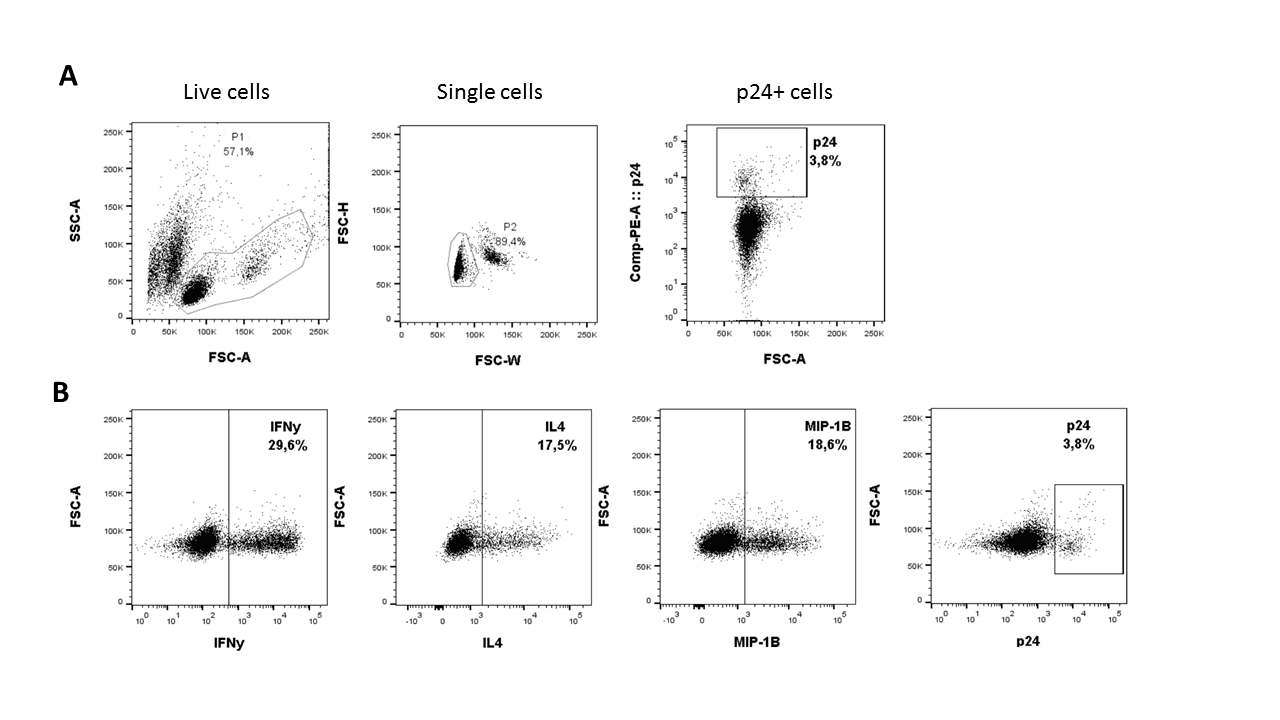

Supplement: S3 Fig — (A) Gating of p24+ cells was performed using a live cell gate using FSC and SSC (left panel), a single cell gate using FSC width (middle panel) and a p24+ cell gate (right panel). (B) Single staining’s for IFNγ (left), IL-4 (2nd left), MIP-1α (3rd left) and p24 (right) of T cells re-stimulated with PMA and ionomycin for 6hrs in the presence of Brefeldin A. Markers are set on positive cells and used for subsequent analysis of T-cell phenotype. (TIF) [file ppat.1007924.s003.tif]

## Slide 1
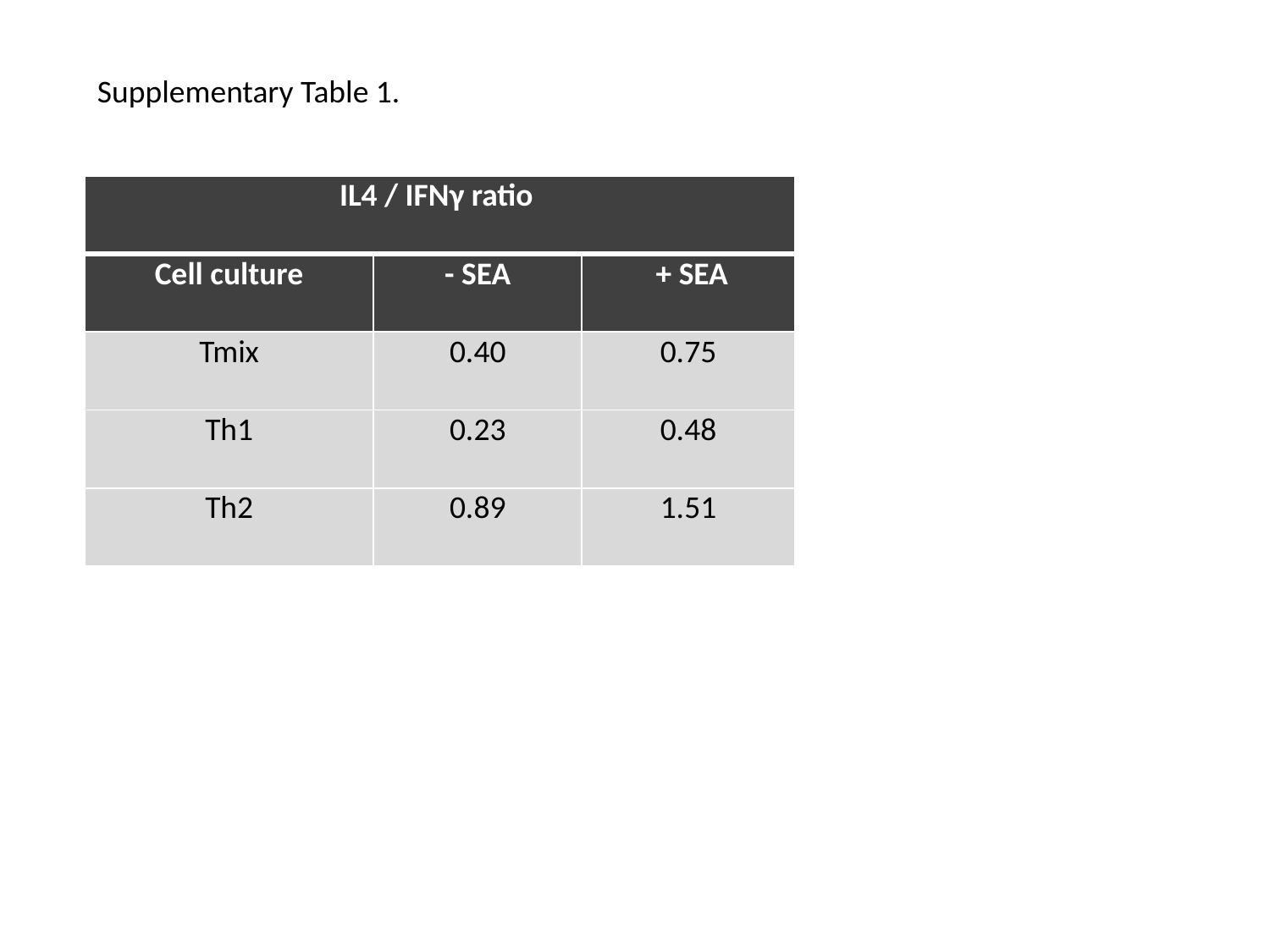

Supplementary Table 1.
| IL4 / IFNγ ratio | | |
| --- | --- | --- |
| Cell culture | - SEA | + SEA |
| Tmix | 0.40 | 0.75 |
| Th1 | 0.23 | 0.48 |
| Th2 | 0.89 | 1.51 |

Supplement: S1 Table — Here the ratio of IL-4 and IFN-γ for each cell culture induced DCs matured in the absence or presence of SEA is demonstrated. (PPTX) [file ppat.1007924.s004.pptx]
